# Antiplatelet activity of a Korean red ginseng–derived saponin fraction and its inhibition of influenza A virus–induced thrombosis

**DOI:** 10.1016/j.jgr.2026.100981

**Published:** 2026-01-21

**Authors:** Ga Hee Lee, Jueun Oh, Jin Pyo Lee, Na Yoon Heo, SangJoon Lee, Dong-Ha Lee

**Affiliations:** a Department of Biomedical Laboratory Science, Namseoul University, Cheonan, 31020, Republic of Korea; b Department of Biological Science, Ulsan National Institute of Science and Technology (UNIST), Ulsan, 44919, Republic of Korea; c Molecular Diagnostics Research Institute, Namseoul University, Cheonan, 31020, Republic of Korea

**Keywords:** Saponins, Ginseng, Platelet aggregation, Thrombosis, Influenza A virus

## Abstract

**Background:**

Platelets play a central role in thrombus formation, which is a major cause of morbidity and mortality worldwide. Viral infections have been reported to further promote thrombus formation, posing a critical risk in unpredictable pandemic situations. Therefore, we evaluated whether a Korean red ginseng–derived saponin fraction could serve as a safe and effective antithrombotic agent by assessing its inhibitory effects.

**Methods:**

Human platelets were examined using an aggregometer, flow cytometry, fluorescence assays, ELISA kits, and western blotting to assess cGMP, intracellular Ca^2+^ levels, fibrinogen binding, granule secretion (ATP and serotonin), and phosphorylation of IP_3_R, VASP, MAPKs, PI3K/Akt, and cPLA_2_. Thrombin-induced clot retraction was quantified. The in vivo effects were further evaluated in influenza A virus (IAV)-infected mice using a FeCl_3_-induced carotid artery thrombosis model, where thrombus formation and blood flow were monitored.

**Results:**

The saponin fraction markedly inhibited platelet aggregation, enhanced cGMP production, and increased phosphorylation of IP_3_R and VASP Ser^239^. Conversely, it suppressed phosphorylation of MAPKs (JNK and p38), PI3K/Akt, and cPLA_2_, thereby blocking downstream signaling pathways. In vivo, IAV infection accelerated thrombus formation and reduced blood flow, whereas administration of the saponin fraction significantly attenuated these pathological changes.

**Conclusion:**

The saponin fraction effectively suppressed platelet activation in vitro and thrombus formation in vivo, even under virus-induced prothrombotic conditions. These findings suggest that the saponin fraction has potential as a safe and effective natural antithrombotic agent.

## Graphical abstract

**Figure:**
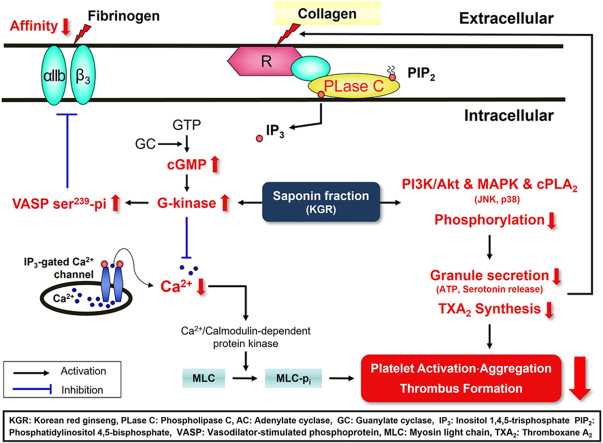

## 1 Introduction

Thrombosis is a major cause of cardiovascular and cerebrovascular diseases, representing a critical global health burden with high morbidity and mortality rates [1]. Once a thrombus forms within the vasculature, blood flow can be obstructed or completely blocked, leading to life-threatening events such as myocardial infarction or stroke [2]. According to the World Health Organization (WHO), approximately 85 % of cardiovascular-related deaths in 2019 were attributable to ischemic events including myocardial infarction and stroke [3]. Although various synthetic antiplatelet drugs are clinically available, their use is often associated with adverse effects such as gastritis and gastrointestinal bleeding. Therefore, natural product–derived agents have attracted attention as potential alternatives [4].

In addition, accumulating evidence indicates that viral infections exacerbate thrombus formation, increasing the risk of severe thrombosis and mortality [5]. This concern has been further highlighted during the recent pandemic [6]. For example, in Sweden, the absolute risks of thromboembolic events among patients with coronavirus disease 2019 (COVID-19) were reported as 0.039 % (401 cases) for deep vein thrombosis, 0.17 % (1761 cases) for pulmonary embolism, and 0.101 % (1002 cases) for major bleeding [7]. These findings suggest that virus-associated thrombotic complications prolong hospitalization, increase healthcare costs, and raise mortality risk. Consequently, there is an urgent need to develop targeted therapeutic strategies to mitigate infection-related thrombosis.

Platelets serve a key function in the onset of thrombus formation. Although prompt activation after vessel injury is required for hemostasis, overactivation or loss of control promotes thrombosis and endangers patients. Therefore, clarifying the mechanistic basis of platelet activation and pinpointing the regulatory circuits is fundamental to designing effective preventive and therapeutic approaches to thrombotic disease.

Mechanistically, platelet activation is initiated through hydrolysis of phosphatidylinositol 4,5-bisphosphate (PIP_2_) by phospholipase C (PLC), producing inositol 1,4,5-trisphosphate (IP_3_) and diacylglycerol (DAG) [8]. IP_3_ binds to its receptor (IP_3_R) located on the endoplasmic reticulum (ER) membrane, thereby opening calcium channels and releasing stored calcium into the cytosol [8,9]. The resultant rise in cytosolic calcium triggers platelet activation [8]. Subsequently, phosphorylation of mitogen-activated protein kinases (MAPKs) such as Erk, JNK, and p38 occurs, leading to secretion of granule-derived mediators including ATP and serotonin, which further amplify platelet activation through autocrine and paracrine signaling [10]. In parallel, activation of the phosphoinositide 3-kinase (PI3K)/Protein kinase B (Akt) pathway promotes granule secretion and integrin αIIbβ_3_ conformational changes, thereby enhancing fibrinogen binding and platelet aggregation [11]. Both MAPK and PI3K/Akt cascades also stimulate arachidonic acid (AA) metabolism and thromboxane A_2_ (TXA_2_) generation, which further potentiate platelet activation and vasoconstriction [11,12].

Conversely, endothelium-derived factors such as prostaglandin I_2_ (PGI_2_) and nitric oxide (NO) act as negative regulators of platelet function. PGI_2_ and NO elevate intracellular cyclic adenosine monophosphate (cAMP) and cyclic guanosine monophosphate (cGMP), respectively, thereby attenuating platelet aggregation [13]. These cyclic nucleotides activate protein kinase A (PKA) and protein kinase G (PKG), which phosphorylate IP_3_R and associated regulatory proteins, ultimately suppressing calcium release into the cytosol [14]. Moreover, phosphorylation of vasodilator-stimulated phosphoprotein (VASP) reduces the binding affinity between integrin αIIbβ_3_ and fibrinogen, with Ser^157^ phosphorylation being cAMP-dependent and Ser^239^ phosphorylation being cGMP-dependent [14]. Alongside these intrinsic pathways, increasing interest has been directed toward plant-derived natural compounds with potential antiplatelet activity [15].

Korean red ginseng contains a complex saponin fraction rich in ginsenosides such as Rg1, Rb1, and Rc. Accumulating evidence indicates that these compounds possess a broad spectrum of pharmacological activities, including anti-inflammatory, antioxidant, and immunomodulatory actions. [16,17]. More recently, the antiplatelet properties of ginseng-derived saponins have been highlighted, particularly in the context of platelet activation and aggregation [[18], [19], [20], [21]]. However, most prior studies on ginseng-derived saponins have been conducted under non-infectious conditions, primarily focusing on thrombin-induced platelet activation [[18], [19], [20], [21]]. In contrast, the present study aimed to evaluate the antiplatelet effects of ginseng saponin fractions under collagen-stimulated platelet activation in a virus-associated environment, a condition that has not been sufficiently addressed in previous studies.

While thrombin stimulation predominantly serves as a potent amplifier of platelet activation during the later stages of thrombus formation, collagen is the first subendothelial matrix component exposed upon vascular endothelial injury, and collagen-mediated platelet activation represents a critical initiating event in the early phase of thrombogenesis [22,23].

In addition, viral infections are increasingly recognized as prothrombotic conditions that induce inflammatory responses and endothelial dysfunction, thereby promoting platelet hyperreactivity and thrombus formation [24,25]. Nevertheless, whether the antiplatelet effects of ginseng saponin fractions are preserved during collagen-mediated platelet activation under virus-associated prothrombotic conditions remains largely unexplored.

Therefore, the present study aimed to elucidate the inhibitory mechanisms of ginseng saponin fractions on collagen-induced platelet activation and, importantly, to determine whether these effects are sustained in a virus-associated thrombotic environment. Through this approach, we sought to evaluate the potential of ginseng saponin fractions as therapeutic candidates for mitigating infection-related thrombosis.

## 2 Materials and methods

A comprehensive description of the chemical reagents and experimental procedures, including the animal handling, preparation of human and rat platelets, assessment of in vitro parameters, and in vivo study protocols, is provided in the Supplementary Material and Methods.

## 3 Results

### 3.1 Reduction of platelet aggregation by increasing concentrations of saponin fraction

**Fig. 1:**
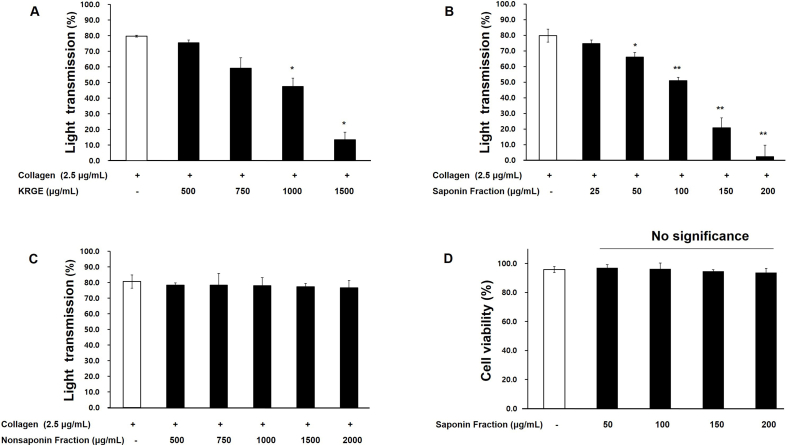
Effects of Korean red ginseng extract (KRGE), saponin fraction, and nonsaponin fraction on collagen-induced platelet aggregation. (A) Inhibition of platelet aggregation by KRGE. (B) Inhibition of platelet aggregation by the saponin fraction. (C) No inhibitory effect of the nonsaponin fraction. (D) Platelet viability after treatment with the saponin fraction. Data are expressed as mean ± SD (n = 4). Statistical significance is indicated as ∗p < 0.05, ∗∗p < 0.01 compared with collagen-stimulated platelets.

Korean red ginseng extract (KRGE) inhibited platelet aggregation in a concentration-dependent manner. When platelets were stimulated with collagen (2.5 μg/mL) alone, the aggregation rate was 79.7 %, which decreased to 13.5 % at 1500 μg/mL of KRGE (Fig. 1A). To identify the major constituents responsible for the antiplatelet activity of KRGE, saponin and nonsaponin fractions were separately tested. The saponin fraction exerted a concentration-dependent inhibitory effect on platelet aggregation (Fig. 1B), whereas the nonsaponin fraction showed no effect at any concentration, displaying aggregation rates similar to those of the control (Fig. 1C).

Specifically, collagen alone resulted in 79.8 % light transmittance, while the saponin fraction at 25, 50, 100, 150, and 200 μg/mL reduced platelet aggregation to 74.8 %, 66.0 %, 51.0 %, 20.8 %, and 2.3 %, respectively (Fig. 1B). Cytotoxicity assays revealed that the saponin fraction did not reduce cell viability, showing values comparable to or even higher than the DMSO control (Fig. 1D). These findings indicate that the antiplatelet effect of KRGE is primarily attributable to the saponin fraction, which is non-toxic to platelets.

### 3.2 Increased cGMP levels lead to enhanced IP_3_R phosphorylation, reduced intracellular calcium, increased VASP Ser^239^ phosphorylation, and inhibition of integrin αIIbβ_3_–Fibrinogen binding

**Fig. 2:**
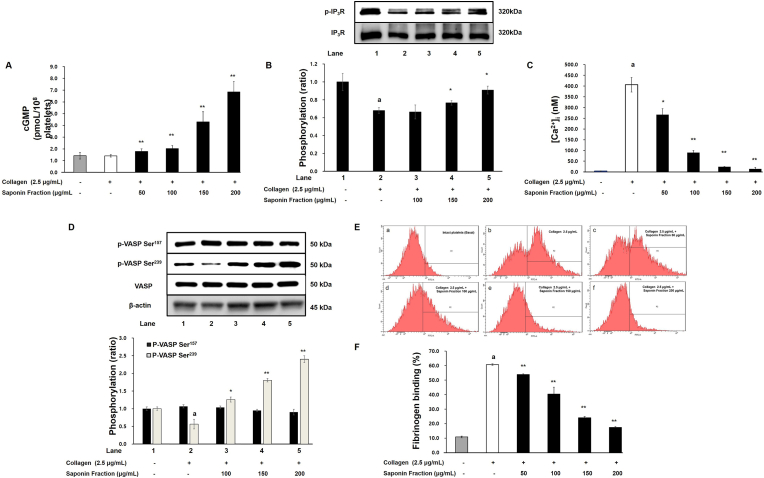
Effects of the saponin fraction on cGMP production and downstream signaling in collagen-stimulated platelets. (A) Increase in cGMP levels by saponin fraction treatment. (B) Increase in IP_3_R phosphorylation. (C) Reduction of intracellular Ca^2+^ mobilization. (D) Increase in VASP Ser^239^ phosphorylation and no change in Ser157 phosphorylation. (E) Inhibition of fibrinogen binding to integrin αIIbβ_3_. Data are expressed as mean ± SD (n = 4). Statistical significance is indicated as ∗p < 0.05, ∗∗p < 0.01 compared with collagen-stimulated platelets.

One of the major mechanisms underlying platelet inhibition is the elevation of cyclic nucleotides. Under collagen stimulation, treatment with the saponin fraction (50, 100, 150, 200 μg/mL) significantly increased intracellular cGMP levels in a concentration-dependent manner, whereas cAMP levels remained unchanged (Fig. 2A). Concomitantly, phosphorylation of the IP3 receptor (IP_3_R), a calcium channel, was enhanced in a concentration-dependent manner upon saponin fraction treatment (Fig. 2B). Furthermore, intracellular calcium mobilization ([Ca^2+^]i) was markedly reduced (Fig. 2C).

Analysis of downstream signaling showed that phosphorylation of VASP at Ser^157^ (cAMP-dependent) was unchanged, while phosphorylation at Ser^239^ (cGMP-dependent) increased in a concentration-dependent manner (Fig. 2D). This finding is consistent with the observed rise in cGMP (Fig. 2A), indicating that the saponin fraction acts specifically through the cGMP pathway. Additionally, the saponin fraction reduced collagen-induced integrin αIIbβ_3_–fibrinogen binding from 60.7 ± 0.5 % to 17.3 ± 0.6 % (Fig. 2E and F). Taken together, these results suggest that the saponin fraction inhibits platelet aggregation by activating the cGMP signaling pathway, leading to enhanced IP_3_R phosphorylation, reduced intracellular calcium release, and increased VASP Ser^239^ phosphorylation, thereby suppressing integrin αIIbβ_3_–fibrinogen interactions.

### 3.3 Inhibition of MAPK (JNK and p38) and PI3K/Akt pathways reduces granule secretion, while suppression of cPLA_2_ phosphorylation decreases TXA_2_ synthesis

**Fig. 3:**
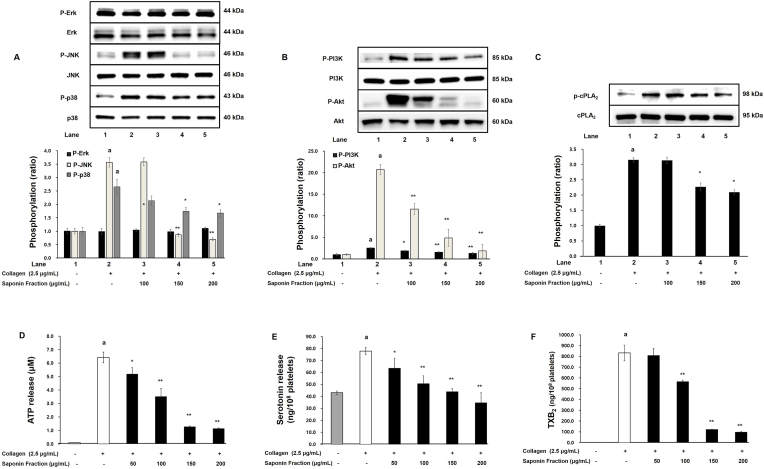
Inhibition of MAPK, PI3K/Akt, and cPLA_2_ phosphorylation and effects on downstream signaling in collagen-stimulated platelets. (A) Changes in MAPK phosphorylation showing inhibition of JNK and p38 with no significant effect on Erk. (B) Inhibition of PI3K and Akt phosphorylation. (C) Inhibition of cPLA_2_ phosphorylation. (D) Reduction of ATP release. (E) Reduction of serotonin release. (F) Reduction of TXB_2_ levels (indicator of TXA_2_ production). Data are expressed as mean ± SD (n = 4). Statistical significance is indicated as ∗p < 0.05, ∗∗p < 0.01 compared with collagen-stimulated platelets.

Since MAPK and PI3K/Akt phosphorylation are known to regulate platelet granule secretion, the effects of the saponin fraction were examined. Collagen stimulation increased phosphorylation of MAPK family proteins; however, phosphorylation of JNK and p38, but not Erk, was significantly reduced by saponin fraction treatment (Fig. 3A). Phosphorylation of PI3K and Akt was also dose-dependently decreased (Fig. 3B).

Consistent with these signaling changes, collagen-induced release of ATP and serotonin from platelet granules was significantly inhibited by the saponin fraction in a concentration-dependent manner (Fig. 3D and E). This suggests that suppression of MAPK (JNK and p38) and PI3K/Akt phosphorylation contributes to inhibition of secondary platelet activation via blockade of granule secretion.

Furthermore, collagen-induced phosphorylation of cPLA_2_, which releases AA for TXA_2_ synthesis, was attenuated by the saponin fraction (Fig. 3C). Consequently, levels of TXB_2_, a stable metabolite of TXA_2_, were significantly reduced (Fig. 3F). Because TXA_2_ is highly unstable, TXB_2_ levels were measured as an indicator of TXA_2_ production. These results demonstrate that the saponin fraction suppresses both granule secretion and the cPLA_2_–TXA_2_ pathway via inhibition of PI3K/Akt and MAPK (JNK and p38) signaling, thereby contributing to its antiplatelet effect.

### 3.4 Inhibition of thrombin-induced platelet clot retraction

**Fig. 4:**
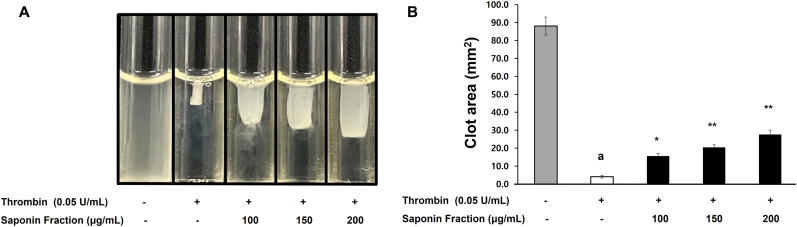
Inhibitory effects of the saponin fraction on thrombin-induced clot retraction in platelets. (A) Representative images showing inhibition of clot retraction. (B) Quantification of clot area (mm^2^). Data are expressed as mean ± SD (n = 4). Statistical significance is indicated as ∗p < 0.05, ∗∗p < 0.01 compared with thrombin-stimulated platelets.

Platelet activation involves intracellular signaling, while secondary thrombus stabilization relies on coagulation factors. Thrombin promotes thrombus stabilization by forming fibrin, leading to firm clot retraction. In this study, treatment with thrombin (0.05 U/mL) induced platelet clot retraction, whereas co-treatment with the saponin fraction markedly reduced clot retraction and resulted in loosening of platelet aggregates (Fig. 4). Quantitative analysis showed that thrombin alone decreased the clot area to 4.8 ± 2.2 mm^2^, whereas treatment with 200 μg/mL of the saponin fraction increased the area to 43.5 ± 2.9 mm^2^, indicating significant inhibition of clot retraction.

### 3.5 Protective effect of saponin fraction against virus-induced thrombosis in the FeCl_3_ carotid artery model

**Fig. 5:**
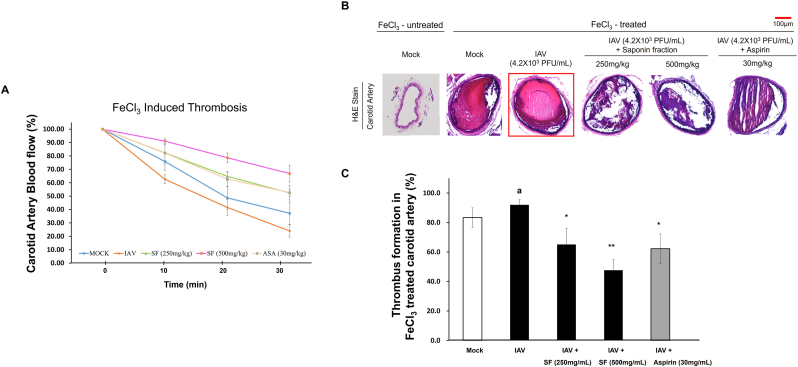
Effects of the saponin fraction on carotid artery blood flow and thrombus formation in the FeCl_3_-induced carotid artery thrombosis mouse model with or without IAV infection. (A) Changes in carotid artery blood flow after FeCl_3_ treatment. Compared with mock controls, IAV-infected mice showed accelerated reduction in blood flow. Oral administration of the saponin fraction (SF) attenuated this reduction. The IAV (4.2 × 10^3^ PFU/mL) + SF 250 mg/kg group showed blood flow comparable to that of the IAV + aspirin (ASA) group, while the IAV + SF 500 mg/kg group showed superior blood flow preservation and thrombus inhibition compared with the IAV + ASA group. (B) H&E-stained carotid artery sections showing increased thrombus formation by IAV infection and its reduction by SF treatment. (C) Quantification of thrombus area (% of lumen occupied by thrombus). Data are expressed as mean ± SD (n = 5). Statistical significance is indicated as ∗p < 0.05, ∗∗p < 0.01 compared with IAV-infected controls.

FeCl_3_ treatment in wild-type mice induced progressive arterial occlusion, as evidenced by a gradual decline in blood flow (Fig. 5A). In influenza A virus (IAV)-infected mice, this reduction in blood flow was markedly accelerated, indicating that viral infection promotes thrombosis. In contrast, oral administration of the saponin fraction effectively suppressed the decline in blood flow. Remarkably, treatment with 500 mg/kg saponin fraction maintained stable blood flow, even more effectively than the positive control aspirin group (ASA), while 250 mg/kg saponin fraction exerted effects comparable to aspirin (Table 2).

Histological analysis by H&E staining supported these findings (Fig. 5B and C). In IAV-infected mice, dense thrombi completely filled the vascular lumen compared to the Mock group. Treatment with 250 mg/kg saponin fraction significantly reduced thrombus formation to levels similar to aspirin, whereas 500 mg/kg almost completely prevented thrombus formation (Fig. 5B and C).

These results suggest that if adequate plasma concentrations can be achieved pharmacologically, the saponin fraction may serve as a safe antithrombotic candidate with potentially lower bleeding risks compared to aspirin.

## 4 Discussion

Korean red ginseng has long been reported to exert antiplatelet and antithrombotic effects; however, the specific active constituents mediating these effects have not been clearly defined [16,17]. In this study, we hypothesized and verified that ginsenosides, the major bioactive components of ginseng, play a pivotal role in inhibiting platelet function. Unlike previous studies that primarily examined the effects of saponins under thrombin stimulation, we extended the scope and significance of the research by employing collagen, a more physiologically relevant agonist [[18], [19], [20], [21]]. Furthermore, inspired by recent reports that viral infections accelerate thrombosis, we investigated whether the saponin fraction retains antithrombotic activity under pathological conditions in vivo [5,6]. This approach is valuable as it not only elucidates the molecular mechanisms of saponins but also explores their therapeutic potential in disease contexts.

Our results clearly demonstrated that the saponin fraction purified from Korean red ginseng (SF) significantly inhibited platelet aggregation, whereas the nonsaponin fraction did not. This strongly suggests that the antiplatelet activity of red ginseng is attributable to its saponin constituents. The saponin fraction contains various ginsenosides, including Rb1, Rb2, Rc, Rd, Rg3, Re, Rg1, and Rg2, some of which have been previously reported to exert antiplatelet effects [26,27]. Therefore, rather than a single compound, the synergistic action of multiple ginsenosides appears to mediate the observed inhibitory effects, consistent with our findings.

Mechanistically, the most notable observation was that the saponin fraction selectively increased cGMP levels under collagen stimulation. This is likely mediated by activation of guanylate cyclase (GC) or inhibition of phosphodiesterase (PDE) [28,29]. Elevated cGMP in turn activated PKG signaling, as evidenced by increased phosphorylation of VASP at Ser^239^, enhanced IP_3_R phosphorylation, and suppression of intracellular calcium mobilization. Downstream, these events resulted in reduced binding affinity of integrin αIIbβ_3_ to fibrinogen, thereby attenuating platelet aggregation.

Additionally, the saponin fraction suppressed phosphorylation of MAPK (JNK and p38) and PI3K/Akt pathways, leading to inhibition of ATP and serotonin release and reduced integrin activation. No changes were observed in Erk signaling, consistent with prior reports that collagen predominantly activates JNK and p38 [30]. Although PI3K activation is transient and baseline activity was low, significant inhibition was evident at the level of downstream Akt [31]. Importantly, both p38 MAPK and PI3K/Akt pathways regulate cPLA_2_ phosphorylation, which drives AA release and subsequent TXA_2_ synthesis [26,32,33]. The saponin fraction inhibited this process as well, further contributing to the multifaceted blockade of platelet activation.

These inhibitory effects were not restricted to primary aggregation events but extended to secondary hemostatic processes, including thrombin-induced fibrin formation and clot retraction. This suggests that saponins may modulate platelet function at multiple stages of thrombosis. Clinically, ingestion of ginseng extracts containing saponins has been associated with reduced collagen-induced platelet aggregation and blood coagulation [34,35]. Moreover, individual ginsenosides such as Rg1, Rg2, and Rg3 have been independently confirmed to exert antiplatelet effects [[36], [37], [38]]. Notably, Korean red ginseng contains higher levels of Rg2 and Rg3 compared to American ginseng, which may account for its superior antiplatelet and antithrombotic efficacy [39]. This is well supported by the fact that the contents of Rg1, Rg2, and Rg3 in the SF used in this study were high at 96.51 ± 1.40 mg/g-SF (Table 1).

**Table 1:** Contents of eleven ginsenosides in SF.

Ginsenosides	Contents (mg/g-SF)
Rg1	24.72 ± 0.38
Re	29.60 ± 0.40
Rf	21.42 ± 0.50
Rh1	21.42 ± 0.50
Rg2s	23.07 ± 0.29
Rb1	108.15 ± 0.87
Rc	44.84 ± 0.80
Rb2	40.60 ± 0.46
Rd	20.08 ± 0.37
Rg3s	34.14 ± 0.51
Rg3r	14.61 ± 0.19
Sum	382.65 ± 4.88

SF, Saponin fraction from Korean red ginseng.

Contents, concentration of ginsenoside in 1g-SF.

**Table 2:** Effects of Saponin fraction of Korean red ginseng (SF) on blood flow of FeCl_3_-induced thrombosis model.

Treatment	Dose (mg/kg)	Blood flow rate (%)		
		10min	20min	30min
Mock	0	75.87 ± 6.80	48.67 ± 8.38	37.20 ± 8.34
IAV	0	62.62 ± 3.26	41.54 ± 5.93	23.99 ± 4.66^#^
SF + IAV	250 mg/kg	82.28 ± 6.39∗∗	64.79 ± 3.19∗∗∗	52.32 ± 2.39∗∗∗
	500 mg/kg	91.10 ± 1.62∗∗∗	78.62 ± 3.55∗∗∗	66.85 ± 6.34∗∗∗
Aspirin + IAV	30 mg/kg	77.36 ± 11.72	65.22 ± 2.83∗∗	48.04 ± 10.27∗∗

Each group of rats was orally administered SF and aspirin at each concentration for 7 days prior to FeCl_3_ exposure, and the results at 10, 20, and 30 min after FeCl_3_ exposure were presented (n = 5). #p < 0.05 from MOCK (Normal control). ∗p < 0.05, ∗∗p < 0.01, and ∗∗∗p < 0.001 from IAV group.

Importantly, the present study demonstrated that the antiplatelet and antithrombotic activities of the saponin fraction are preserved even under conditions of viral infection. Viral infections are known to induce innate immune activation and the release of pro-inflammatory cytokines, which subsequently lead to endothelial activation or dysfunction, thereby promoting thrombosis [40]. Such endothelial alterations are accompanied by a reduction in antiplatelet mediators, including nitric oxide (NO) and prostacyclin (PGI_2_), along with increased expression of procoagulant and adhesive signals such as tissue factor (TF) and von Willebrand factor (vWF) [41]. Collectively, these changes establish a thrombosis-prone environment characterized by heightened platelet hyperreactivity and amplified coagulation. These pathological processes are defined as thromboinflammation or immunothrombosis and have been particularly emphasized in coronavirus-associated thrombosis [42]. Meanwhile, infection with influenza A virus (IAV), which was employed in the present study, has likewise been well established to induce platelet activation and thrombosis through comparable inflammatory and endothelial mechanisms [43].

Within this conceptual framework, IAV infection significantly accelerated thrombus formation in the FeCl_3_-induced carotid artery thrombosis model. In contrast, oral administration of the saponin fraction restored carotid blood flow and effectively suppressed thrombus formation. Notably, a dose of 250 mg/kg exhibited antithrombotic efficacy comparable to that of aspirin, while a dose of 500 mg/kg produced an even more pronounced inhibitory effect.According to previous studies, respiratory viruses can directly activate platelets through virus–platelet interactions or receptor-mediated signaling involving molecules such as integrin αIIbβ_3_, or alternatively converge on platelet activation programs indirectly via inflammatory responses and endothelial pathways [5]. In addition, certain viruses, including SARS-CoV-2, have been reported to induce platelet activation through intracellular signaling cascades such as MAPK phosphorylation or activation of the PI3K/Akt pathway [44,45].

Consistent with these observations, our in vitro findings demonstrated that the saponin fraction inhibited key platelet activation signaling pathways, including PI3K/Akt and MAPK, while reducing fibrinogen binding to integrin αIIbβ_3_ and granule secretion, thereby effectively suppressing platelet aggregation. Furthermore, ginseng-derived saponin components have been proposed in previous studies as candidate agents capable of counteracting thrombosis associated with infection by SARS-CoV-2, another respiratory virus, through modulation of platelet activation and coagulation pathways [25].

Therefore, unlike most previous studies that evaluated the antiplatelet and antithrombotic effects of saponins under non-infectious or simplified stimulation conditions, the present study demonstrates in an in vivo model that the antithrombotic efficacy of the saponin fraction is maintained even within a virus-induced prothrombotic environment. In particular, the effective suppression of thrombus formation and restoration of blood flow under the pathological condition of influenza A virus infection highlight the saponin fraction as a promising candidate with inhibitory potential against virus-associated thrombosis.

In conclusion, the saponin fraction activated the cGMP–PKG pathway under collagen stimulation, leading to suppression of intracellular calcium signaling and integrin activity, while simultaneously inhibiting MAPK and PI3K/Akt signaling, thereby reducing granule release, TXA_2_ synthesis, and clot retraction. These actions were maintained not only under physiological conditions but also in a pathological context of IAV-induced thrombosis. Collectively, this study expands beyond thrombin-based investigations to collagen-based stimulation and viral infection models, highlighting the potential of Korean red ginseng saponin fraction as a natural antithrombotic candidate.
