# Supplementary material for: Antiplatelet activity of a Korean red ginseng–derived saponin fraction and its inhibition of influenza A virus–induced thrombosis
Source: J Ginseng Res. 2026 Jan 21;50(3):100981. doi: 10.1016/j.jgr.2026.100981 (PMC13149486; doi:10.1016/j.jgr.2026.100981)
Supplement: Multimedia component 1 [file mmc1.docx]

**Supplementary Material and Methods**

Antiplatelet activity of a Korean red ginseng–derived saponin fraction and its inhibition of Influenza A virus–induced thrombosis

Ga Hee Lee^1†^, Jueun Oh^2†^, Jin Pyo Lee^1^, Na Yoon Heo^1^, SangJoon Lee^2*^ and Dong-Ha Lee^1,3*^

^1^Department of Biomedical Laboratory Science, Namseoul University, Cheonan 31020, Republic of Korea

^2^Department of Biological Science, Ulsan National Institute of Science and Technology (UNIST), Ulsan 44919, Republic of Korea

^3^Molecular Diagnostics Research Institute, Namseoul University, Cheonan 31020, Republic of Korea

^†^These authors are contributed equally to paper.

* Correspondence: dhlee@nsu.ac.kr (D.H.L.), sangjoon.lee@unist.ac.kr (S.J.L.)

Tel: +82-41-580-2148, Fax:+82-42-580-2932

E-mail: [dhlee@nsu.ac.kr](mailto:dhlee@nsu.ac.kr)

2.1. Reagents

Antibodies and lysis buffer for Western blotting were obtained from Cell Signaling Technology (Beverly, MA, USA). Korean red ginseng extract (KRGE), saponin fraction, and nonsaponin fraction were kindly provided by the Korea Ginseng Corporation (Gwachon, Korea). Platelet agonists were purchased from Chrono-Log Corporation (Havertown, PA, USA). Fura-2AM and Alexa Fluor 488–conjugated fibrinogen were obtained from Invitrogen Molecular Probes (Eugene, OR, USA). Enzyme immunoassay kits for thromboxane B₂ (TXB₂), serotonin, ATP, and cyclic nucleotides (cAMP/cGMP) were purchased from Cayman Chemical Co. (Ann Arbor, MI, USA). Polyvinylidene difluoride (PVDF) membranes and enhanced chemiluminescence (ECL) reagents were supplied by Thermo Fisher Scientific (Seoul, Korea). Unless otherwise specified, all other chemicals were purchased from Sigma-Aldrich (St. Louis, MO, USA).

2.2. Preparation of substances derived from Korean red ginseng

Fresh ginseng roots were prepared and processed by steaming and drying to make red ginseng in red ginseng manufacturing factory of Korea Ginseng Corporation (Buyeo, Chung-nam, Korea). Washed fresh ginseng roots were steamed for 4 hours while slowly raising its temperature from 50 ℃ to 98 ℃ and then firstly dried at 60~70 ℃ for 15 hours. Thereafter, secondary drying process was performed in a closed chamber at 50℃ for 5 days to resulting the red ginseng roots (RG). Red ginseng powder (RGP) was made by finely grinding red ginseng roots prepared as above. The red ginseng roots were also extracted by supercritical extraction, filtered, concentrated and purified to be suitable for red ginseng oil (RGO). To prepare red ginseng extract(KRGE), the root were sequentially extracted 7 times at 87℃ for 12 hours with distilled water. The extracted water was combined followed by filtering and concentrating process. Non-saponin fraction and saponin fraction of the red ginseng were prepared by adsorption chromatography as methods below with Dion HP20 (Mitsubishi Chemical Industries, Ltd.) using red ginseng extract. The red ginseng extract was diluted to 10% in water and then filtered. The diluted solution was subjected to HP20 resin for adsorption then eluted using water, 30% ethanol in water, and 95% ethanol in water, sequentially. The first two fractions (H2O, 30% EtOH in water) were combined, concentrated and spray dried to produce the non-saponin fraction. The last fraction (95% EtOH in water) was concentrated and spray dried to produce the saponin fraction.

2.3. Analysis of ginsenosides in the saponin fraction of Korean red ginseng using UPLC-PDA

A half gram of Red ginseng extract was weighed in a volumetric flask, and 10㎖ of 70% MeOH was added. The extraction was performed in an ultrasonic cleaner (60Hz, Wiseclean, Seoul, Korea) for 30 min. After ultrasonic extraction, entrifugal sepration (Legand Mach 1.6R; Thermo, Frankfurt, Germany) was performed for 10 min at 30,000 rpm. Then, the solution was filtered(0.2 ㎛; Acrodisk, Port Washington, NY, USA) and injected into the UPLC system. The instrumental analysis was performed by a Waters ACQUITY UPLC system (Waters, Millford, MA, USA) composed of a binary solvent manager, sample manager and photo diode array detector (PDA). The chromatographic separation was accomplished on a ACQUITY BEH C18 column (50 ㎜×2.1 ㎜, 1.7 ㎛; Waters). The column temperature was 40°C. The binary gradient elution system consisted of deionized water (A) and acetonitrile (B). The separation was achieved using the following gradient program: 0.5-14.5 min(15-30% B), 14.5-15.5 min(30-32% B), 15.5-16.5 min (32-40% B), 16.5-17.0 min (40-55% B), 17.0-21.0 min (55-90% B), 21-25min (90-15% B), 25-27 min (15%B). The flow rate was set at 0.6 ㎕/min and the sample injection volume was 2.0 ㎕. The three ginsenosides were detected by PDA at 203 nm [1].

2.4. Preparation of Platelet Suspensions

Human platelet-rich plasma (PRP) was sourced from healthy donors via the Korean Red Cross Blood Center (KRBC, Suwon, Korea) after written informed consent was obtained. KRBC processed and released specimens under the Korean Blood Management Act. The study was approved by the Namseoul University Institutional Review Board (IRB; approval No. 1041479-BR-202410-001-01). PRP was spun at 3,000 rpm for 5 min, and platelets were washed in a suspending buffer (pH 7.4) containing 138 mM NaCl, 2.7 mM KCl, 5.5 mM glucose, 0.36 mM NaH₂PO₄, 12 mM NaHCO₃, and 0.49 mM MgCl₂; the pH was adjusted with 1 M HCl. After centrifugation, the platelet pellet was resuspended to a final density of 1 × 10⁸ cells/mL. All steps in this section were performed at room temperature unless otherwise specified, following a previously described method [2].

2.5. Platelet Aggregation Assay

Platelet suspensions (1 × 10⁸ cells/mL) were preincubated with varying concentrations of the saponin fraction (dissolved in dimethyl sulfoxide, DMSO; final DMSO concentration 0.1%). Control groups were treated with the same concentration of DMSO (0.1%). CaCl₂ was then added to a final concentration of 2 mM, and the mixture was stirred at 1000 rpm for 3 min at 37°C. Platelet aggregation was induced by collagen (2.5 µg/mL) and monitored for 5 min using a Chrono-Log aggregometer (Chrono-Log Co., Havertown, PA, USA). Light transmission was used as an indicator of platelet aggregation [3]. KRGE and the nonsaponin fraction were also evaluated under the same conditions.

2.6. Cytotoxicity Assay

LDH leakage served as the cytotoxicity endpoint. Platelet suspensions (1 × 10⁸ cells/mL) were exposed to graded doses of the saponin fraction for 5 min at 37 °C, after which samples were clarified (10,000 × g, 2 min). Supernatant LDH activity was assayed with the Cayman LDH Cytotoxicity kit per the vendor protocol, and absorbance was recorded on a TECAN microplate reader (Salzburg, Austria).

2.7. Measurement of Cyclic Nucleotide (cGMP)

Platelet suspensions (1 × 10⁸ cells/mL) were incubated with the saponin fraction at 37°C for 3 min, followed by stimulation with collagen in the presence of 2 mM CaCl₂. The reaction was terminated after 5 min by adding 1 M HCl. cGMP levels were quantified using enzyme immunoassay kits (Cayman Chemical) and measured with a Synergy HT Multi-Reader (BioTek Instruments, Winooski, VT, USA).

2.8. Measurement of Intracellular Ca²⁺ Mobilization

PRP was incubated with 5 µM Fura-2AM at 37°C for 60 min. Washed platelet suspensions were prepared as described above, treated with 2 mM CaCl₂, and stimulated with collagen for 5 min at 37°C. Fluorescence was measured using an F-7000 spectrofluorometer (Hitachi Instruments, Seoul, Korea) with excitation at 340/380 nm and emission at 510 nm. Intracellular Ca²⁺ concentrations were calculated using the Grynkiewicz equation [4].

2.9. Fibrinogen Binding Assay

Platelet suspensions (1 × 10⁸ cells/mL) were incubated with the saponin fraction and Alexa Fluor 488–conjugated fibrinogen (30 µg/mL) for 5 min at 37°C in the dark. Reactions were fixed with 0.5% paraformaldehyde in cold PBS. Binding of fibrinogen to integrin αIIbβ_3_ was analyzed by flow cytometry (BD Biosciences, San Jose, CA, USA) using CellQuest software.

2.10. Measurement of ATP, Serotonin, and TXA₂ Release

Platelet suspensions were treated with the saponin fraction, followed by incubation with CaCl₂ (final 2 mM) and stimulation with collagen for 5 min at 37°C. Supernatants were collected after centrifugation (10,000 × g, 2 min). ATP release was quantified using an ATP Detection Assay Kit (Cayman Chemical) and measured by luminescence. Serotonin was quantified using a Serotonin ELISA Kit (Abcam, Cambridge, UK), and absorbance was read at 410 nm. TXA₂ levels were determined indirectly as TXB₂ using an ELISA kit (Cayman Chemical) at 410 nm.

2.11. Thrombin-Induced Platelet Clot Retraction

PRP (500 μL) was mixed with 2 mM CaCl₂ and 0.05 U/mL thrombin and incubated at 37 °C for 15 min in polyethylene tubes to minimize surface adhesion. Resulting fibrin clots were photographed with a digital camera, and the two-dimensional clot area (mm²) was quantified in ImageJ (NIH, Bethesda, MD, USA).

2.12. Western Blot Analysis

Platelets received the saponin fraction under the indicated conditions and were subsequently stimulated for 5 min. Cells were solubilized in 1× lysis buffer, and lysates were clarified by centrifugation (10,000 × g, 10 min). Protein content was determined by the BCA method. Aliquots containing 30 µg of protein were resolved by SDS–PAGE and electrotransferred to PVDF membranes. Blots were blocked in 3% BSA for 1 h at room temperature, probed overnight at 4 °C with primary antibodies (1:1000), and then incubated with secondary antibodies (1:2000) for 1 h. Chemiluminescent signals were developed with WesternBright ECL (Advansta, San Jose, CA, USA) and quantified by densitometry using Quantity One (Bio-Rad, Hercules, CA, USA). β-actin served as the loading control.

2.13. FeCl₃-Induced Thrombosis Model in Mice

Animal experiments using C57BL/6N mice were approved by the Animal Experimental Ethics Committee of the Ulsan National Institute of Science and Technology (UNIST) (approval No. UNISTIACUC-24-055). All procedures complied with relevant institutional guidelines and the ARRIVE reporting standards. Female mice (8 weeks old, ~20 g) were obtained from UNIST and randomized into five groups (n = 5 per group):

1. Vehicle control (mock),

2. Virus-infected group (influenza A virus [IAV], 4.2 × 10³ PFU/mL),

3. Virus-infected + aspirin (30 mg/kg, positive control),

4. Virus-infected + saponin fraction (250 mg/kg),

5. Virus-infected + saponin fraction (500 mg/kg).

All test compounds were suspended in 0.5% carboxymethylcellulose (CMC) and orally administered. Control and virus-only groups received vehicle (0.5% CMC) only. Mice were housed individually under controlled environmental conditions (23 ± 2°C, 50 ± 10% humidity, 12 h light/dark cycle) with a one-week acclimatization period.

2.14. Measurement of Blood Flow in the FeCl₃-Induced Carotid Artery Model

C57BL/6N mice were infected with IAV 4 days before blood flow analysis. The test agents (0.5% CMC, aspirin, or saponin fractions) were administered orally once daily for 7 consecutive days. Thirty minutes after the final administration, the mice were anesthetized with intraperitoneal Avertin and maintained on a heating pad at 37 °C. The carotid artery was surgically exposed and placed on a plastic platform. To induce thrombosis, a filter paper saturated with 15% FeCl₃ (15 µL) was applied to the artery for 3 min. Blood flow was then monitored in real time for 30 min using an ultrasonic flow probe.

2.15. Histological Analysis of Carotid Artery Thrombi

After blood flow measurement, the vessels at the FeCl₃-induced site were harvested and fixed in 10% neutral-buffered formalin for 24 hours, followed by paraffin embedding. Paraffin blocks were sectioned at a thickness of 4 μm, mounted on glass slides, and subjected to H&E staining. The tissues were then examined under a light microscope (Leica Microsystems, Germany), and imaging was performed using LAS X software. The ratio of thrombus area to total vessel area was calculated using the ImageJ program (NIH, USA) and used for statistical analysis.

2.16. Statistical Analysis

Data are shown as mean ± standard deviation (SD). Groups were compared by one-way ANOVA, and post hoc comparisons used the Tukey–Kramer procedure. All statistics were run in SPSS 21.0.0.0 (SPSS Inc., Chicago, IL, USA). Differences with p < 0.05 were regarded as significant.

[1] Park HW, In G, Han ST, Lee MW, Kim SY, Kim KT, Cho BG, Han GH, Chang IM. Simultaneous determination of 30 ginsenosides in Panax ginseng preparations using ultra performance liquid chromatography. J Ginseng Res. 2013;37:457–467.

[2] Lee GH, Lee JP, Heo NY, Lee CD, Kim G, Wahab AA, Rhee MH, Lee S, Lee DH. Dioscin from Smilax china rhizomes inhibits platelet activation and thrombus formation via up-regulating cyclic nucleotides. Sci Rep 2025;15:25538.

[3] Ko SN, Son JW, Kim GR, Kim MS, Lee YJ, Kim SJ, Shin JH, Jo DI, Bok WY, Oh HG, Kwon HW. The inhibitory effects of Glycyrrhiza uralensis on human platelet aggregation and thrombus formation. Biomed Sci Lett 2023;29:242–8.

[4] Grynkiewicz G, Poenie M, Tsien RY. A new generation of Ca2+ indicators with greatly improved fluorescence properties. J Biol Chem 1985;260:3440–8.
